# Use of whole-genome sequencing to distinguish relapse from reinfection in a completed tuberculosis clinical trial

**DOI:** 10.1186/s12916-017-0834-4

**Published:** 2017-03-29

**Authors:** Adam A. Witney, Anna L. E. Bateson, Amina Jindani, Patrick P. J. Phillips, David Coleman, Neil G. Stoker, Philip D. Butcher, Timothy D. McHugh, RIFAQUIN Study Team

**Affiliations:** 1 Institute for Infection and Immunity, St George’s University of London, London, UK; 2 UCL Centre for Clinical Microbiology, Royal Free Campus, UCL, London, UK; 3 MRC Clinical Trials Unit at UCL, London, UK

**Keywords:** Whole genome sequencing, Tuberculosis, Clinical trial

## Abstract

**Background:**

RIFAQUIN was a tuberculosis chemotherapy trial in southern Africa including regimens with high-dose rifapentine with moxifloxacin. Here, the application of whole-genome sequencing (WGS) is evaluated within RIFAQUIN for identifying new infections in treated patients as either relapses or reinfections. WGS is further compared with mycobacterial interspersed repetitive units-variable number tandem repeats (MIRU-VNTR) typing. This is the first report of WGS being used to evaluate new infections in a completed clinical trial for which all treatment and epidemiological data are available for analysis.

**Methods:**

DNA from 36 paired samples of *Mycobacterium tuberculosis* cultured from patients before and after treatment was typed using 24-loci MIRU-VNTR, in silico spoligotyping and WGS. Following WGS, the sequences were mapped against the reference strain H37Rv, the single-nucleotide polymorphism (SNP) differences between pairs were identified, and a phylogenetic reconstruction was performed.

**Results:**

WGS indicated that 32 of the paired samples had a very low number of SNP differences (0–5; likely relapses). One pair had an intermediate number of SNP differences, and was likely the result of a mixed infection with a pre-treatment minor genotype that was highly related to the post-treatment genotype; this was reclassified as a relapse, in contrast to the MIRU-VNTR result. The remaining three pairs had very high SNP differences (>750; likely reinfections).

**Conclusions:**

WGS and MIRU-VNTR both similarly differentiated relapses and reinfections, but WGS provided significant extra information. The low proportion of reinfections seen suggests that in standard chemotherapy trials with up to 24 months of follow-up, typing the strains brings little benefit to an analysis of the trial outcome in terms of differentiating relapse and reinfection. However, there is a benefit to using WGS as compared to MIRU-VNTR in terms of the additional genotype information obtained, in particular for defining the presence of mixed infections and the potential to identify known and novel drug-resistance markers.

**Electronic supplementary material:**

The online version of this article (doi:10.1186/s12916-017-0834-4) contains supplementary material, which is available to authorized users.

## Background

Evaluations of drug trials for tuberculosis (TB) are complicated by the fact that a recurrence of disease can either be due to endogenous relapse of disease or to subsequent exogenous infection with a new strain (reinfection). Historically, during the major TB chemotherapy trials of the 1960s to 1980s (reviewed by Fox et al. [1]), it was not possible to differentiate isolates, and all new infections that occurred after the trial conclusion were labelled as relapses.

From the 1980s, a series of genomic-based methods for typing strains of *Mycobacterium tuberculosis* were developed, in particular IS*6110* restriction fragment length polymorphism (RFLP), spoligotyping and mycobacterial interspersed repetitive units-variable number tandem repeats (MIRU-VNTR) typing [2–4]. Some trials therefore began to use molecular methods to differentiate relapses from reinfections. This was initially through IS*6110* RFLP typing [5–7] and then through MIRU-VNTR typing [8], while other trials continued without any differentiation [9].

MIRU-VNTR became the favoured typing approach because it combined reasonable discrimination with a readout that could both be easily measured and be described in a digital form [3]. More recently, whole-genome sequencing (WGS) has enabled the identification of single-nucleotide polymorphism (SNP) differences, thus leading to far greater discrimination in TB epidemiological studies [10–13].

Two groups have recently used WGS to evaluate paired samples, comparing SNP differences between the original infections and new infections following treatment [14, 15]. The study by Bryant et al. [14] was based on an ongoing clinical trial [16] that was being carried out in sub-Saharan Africa, south and east Asia, and central America. Of the 36 paired samples, 33 were found to be highly similar (≤6 SNPs; classed as relapses) and three were highly divergent (≥1306 SNPs; classed as reinfections).

The report by Guerra-Assunção et al. [15] was not based on a clinical trial, but was taken from the Karonga Prevention Study, a long-term population-based programme in Malawi. In this programme, 60 paired samples collected over a 15-year time period were sequenced, and while the authors also found a clear division in SNP numbers between relapses and reinfections, it was not as marked as in the Bryant study. Thus, they classed 46 samples with 0–8 SNP differences as relapses, and 14 with >100 SNP differences as reinfections.

In this study, we performed WGS and analysed SNPs to compare pre- and post-treatment isolates from the completed RIFAQUIN clinical trial [17], a study evaluating high-dose rifapentine with moxifloxacin, carried out in sub-Saharan Africa. Successful sequencing was carried out on 36 pairs of samples of *M. tuberculosis* recovered before treatment and from those patients showing positive cultures at 6 months, and results were compared with MIRU-VNTR data. Our results agree with the general findings from the two studies referred to above, in that the overwhelming majority of secondary cases were classified as relapses. Importantly, WGS was further able to monitor possible epidemiological connections and sample errors during the trial, which were not detected using MIRU-VNTR. Given the added benefit of WGS in this context, we suggest that WGS should be routinely used as the method of choice in such trials.

## Methods

### RIFAQUIN trial

The RIFAQUIN chemotherapy trial, in collaboration with six institutions in southern Africa, has been previously described [17]. Between August 2008 and August 2011, patients with newly diagnosed smear-positive drug-sensitive TB were randomly assigned to one of the following:

**Control regimen**: 2 months of daily ethambutol, isoniazid, rifampicin and pyrazinamide followed by 4 months of daily isoniazid and rifampicin;

**4-month regimen:** Isoniazid replaced by moxifloxacin daily for 2 months followed by 2 months of twice-weekly moxifloxacin and 900 mg rifapentine; or

**6-month regimen**: Isoniazid replaced by moxifloxacin daily for 2 months followed by 4 months of once-weekly moxifloxacin and 1200 mg rifapentine.

Sputum was examined by microscopy and culture at regular intervals for treatment failure or relapse. Patients had up to 18 months of follow-up post randomisation, with the patients recruited last having 12 months of follow-up post-randomisation. Samples from patients with two or more consecutive *M. tuberculosis*-positive cultures after 6 months (or at the end of treatment) were selected for WGS.

### MIRU-VNTR determination and assignment

The 24-loci MIRU-VNTR typing of these isolates was previously described [17]. Briefly, a 10 μL loop was used to pick up a sample of *M. tuberculosis* colonies by sweeping across growth on a Lowenstein–Jenson (LJ) slope. Bacteria were heat-killed and DNA extraction performed using lysozyme and proteinase K digestion followed by phenol-chloroform extraction and ethanol precipitation [18]. The 24 MIRU-VNTR loci were amplified in eight labelled multiplex PCR reactions, and the amplicons sized, with MapMarker 1000 standard (BioVentures, Murfreesboro, TN, USAs), by capillary electrophoresis on the sequencer (3130 Genetic Analyzer, Applied Biosystems, Waltham, MA, USA). Analysis was carried out using the GeneMapper software (Applied Biosystems, Waltham, MA, USA), which assigns alleles based on the customised bin-sets (fragment sizes and dyes) used to define each allele. For some samples there was variable coverage across the MIRU-VNTR loci using the sequencer, so, where possible, any missing loci were confirmed by single-plex PCR with products sized by standard agarose gel electrophoresis. Where possible, paired samples (pre- and post-treatment) from a given patient were run in parallel.

### Whole-genome sequencing

For the WGS, 50 μL, containing at least 250 ng, of genomic DNA from each sample was sheared using the Covaris E220 for a target size of 200 bp (Peak Incident Power: 175; duty factor: 10%; cycle/burst: 200; temperature: <8 °C; time: 120 s). Libraries were prepared from sheared DNA using the NEB DNA Ultra kit in accordance with standard protocol (New England Biolabs, Hitchin, UK). The NEB adapters were substituted for the set described by Kozarewa and Turner [19]. Libraries were quantified using the Qubit High Sensitivity DNA assay and pooled equimolarly (Invitrogen, UK). The pools were subjected to paired-end sequencing carried out on a single lane of the Illumina HiSeq 2500 (v3 chemistry, read length 100 bp). Samples which produced a low yield were re-pooled and sequenced on a single MiSeq run (v2 chemistry, read length 250 bp).

### Sequence analyses

Sequence reads were mapped to the H37Rv reference genome (RefSeq accession: NC_000962) using bwa mem v0.7.3a-r367 [20], alignments were sorted, and duplicates were removed with samtools v0.1.19 [21]. Site statistics were generated using samtools mpileup and variant sites were filtered based on the following criteria: mapping quality above 30, site quality score above 30, at least four reads covering each site with at least two reads mapping to each strand, at least 75% of reads supporting site (DP4) and an allelic frequency of 1. Sites that failed these criteria in any isolate were removed from the analysis. Phylogenetic reconstruction was performed using RAxML v8.2.3 [22] with a General Time Reversible (GTR) model of nucleotide substitution and a Gamma model of rate heterogeneity; branch support values were determined using 1000 bootstrap replicates. Relapse or reinfection calls were made by applying the above filtering criteria to the individual patients’ paired samples. INDELS were identified using samtools mpileup as above, but setting the minimum fraction of gapped reads for candidates to 0.05.

### In silico spoligotyping and sub-lineage typing

Spoligotypes were generated using SpolPred [23]. Sub-lineages were further determined using the presence or absence of a set of 62 lineage-defining SNPs as derived by Coll et al. [24]*.*

### Mixed infections

For each isolate sequence a count of the percentage of reads supporting a variant base at each genome position was plotted. Mixed isolates can be identified by the presence of an extra peak, suggesting the presence of two genotype populations in the sequenced sample. Base calls for the majority and minority strains were separated based on the per cent reads and pseudo-sequences were generated and subsequently included in the phylogenetic reconstruction as above.

## Results

### Samples studied

**Fig. 1:**
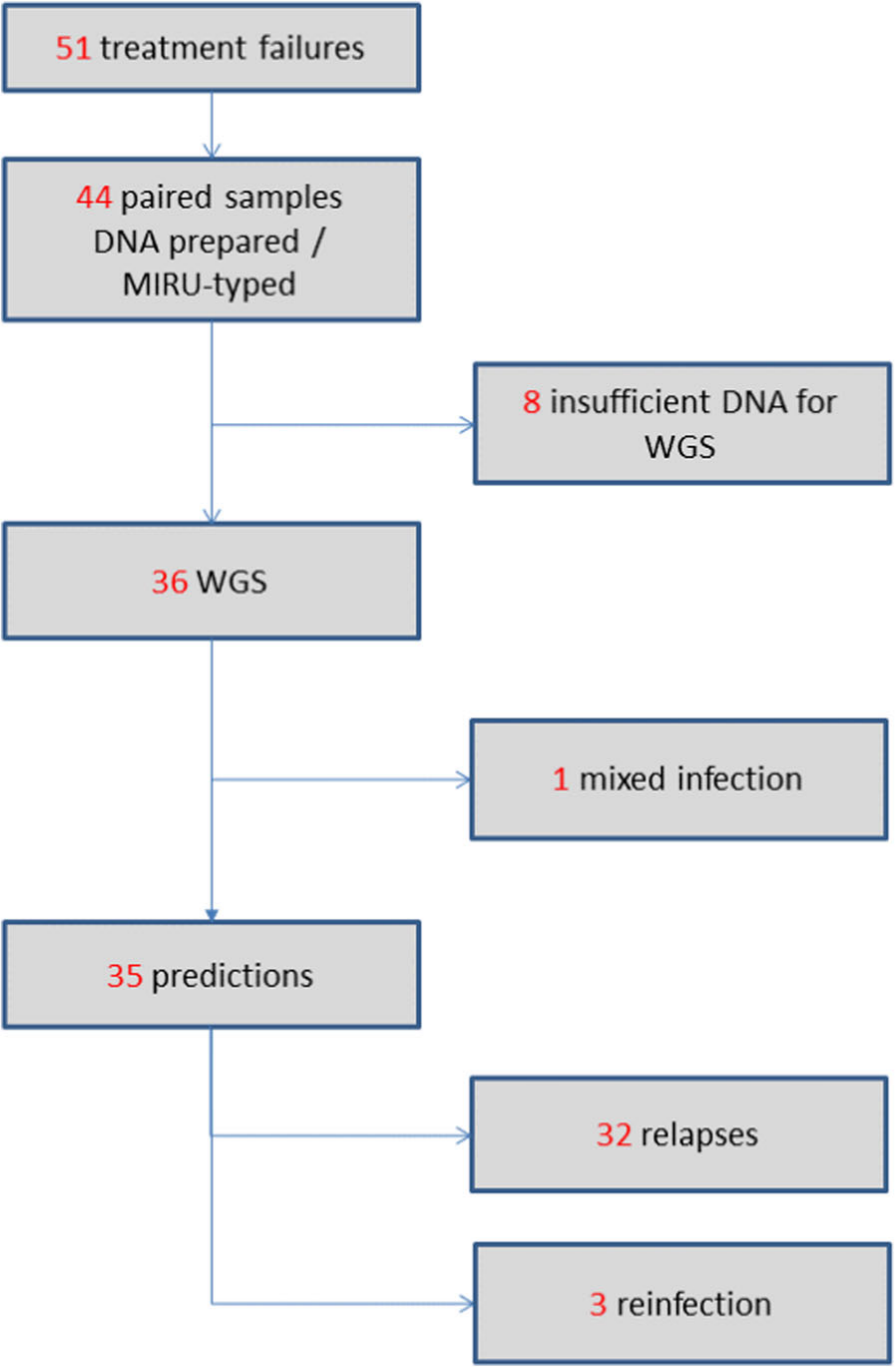
Flowchart of pairs of samples studied. *MIRU* mycobacterial interspersed repetitive units, *WGS* whole-genome sequencing

Figure 1 shows a flowchart of the samples studied. A total of 827 patients, with newly diagnosed, microscopy-positive pulmonary TB were enrolled in South Africa, Zimbabwe, Botswana and Zambia in the trial. Fifty-one patients had positive cultures in post-treatment follow-up and therefore required genotyping to distinguish relapse from reinfection (as per the RIFAQUIN protocol [17]). DNA was available to generate MIRU-VNTR data for 44 pairs of samples (pre- and post-treatment). The remaining DNA was passed for WGS, and good-quality sequences (>20× coverage) were generated for both pre- and post-treatment samples of 36 patients.

SNP differences were determined between the pairs of isolates, and a comparison with MIRU-VNTR differences is shown in Table 1. Two main groups can be identified: 32 pairs of isolates had five or fewer SNP differences, and four pairs of samples had a much higher number of SNP differences (range 737–1329). An additional single pair of isolates differed by 57 SNPs, but this was probably because the pre-treatment isolate contained a mixed infection, as discussed below.

**Table 1:** Comparison between the differences in single-nucleotide polymorphisms and mycobacterial interspersed repetitive units

SNP differences	Number of isolate pairs	MIRU differences
0	19	0 (*n* = 15)^b^, 1 (*n* = 4)
1	7	0 (*n* = 6)^c^, 2 (*n* = 1)
2	1	0
3	2	0
5	3	0^d^
57^a^	1	2
737	1	6
1233	1	0^e^
1294	1	7

*MIRU* mycobacterial interspersed repetitive units, *SNP* single-nucleotide polymorphism

^a^excluded from further SNP analysis as found to be mixed infection, but re-interpreted as a relapse (see text)

^b^two samples <10 loci [2, 7]

^c^one from only two informative loci

^d^ from only five informative loci

^e^from only three informative loci

### Phylogenetic reconstruction of SNPs

**Fig. 2:**
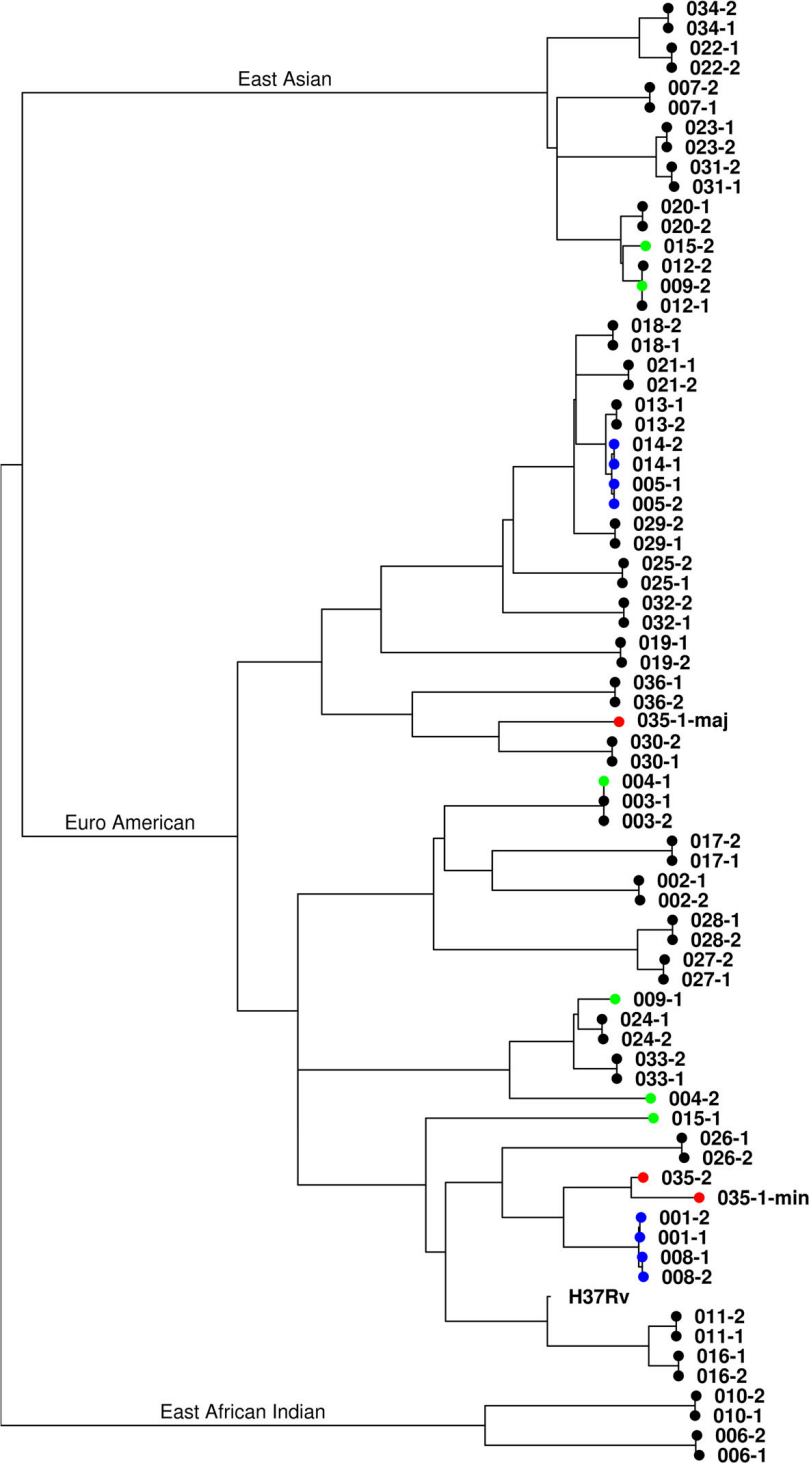
**a** Phylogenetic reconstruction of 36 pairs of isolates. These were inferred using 5132 high-quality single-nucleotide polymorphisms (*SNPs*) following the removal of 661,083 low-quality sites and the remaining invariant sites. The tree was rooted using the H37Rv reference strain sequence. Relapse, reinfection and mixed are denoted with *black/blue*, *green* and *red* tips respectively. *Blue* tip labels are further shown in panels b**–**e. **b**–**e** Branches have been amplified where unexpected similarity was seen; the numbers of SNPs between the most divergent samples are given

Phylogenetic reconstruction of variant SNPs (Fig. 2a) showed that the majority (32 out of 36) of the isolate pairs had low numbers of SNP differences and were therefore clearly determined as cases of relapse. One isolate pair was identified as a mixed infection (see below). The remaining three isolate pairs that had high numbers of SNP differences appear quite divergent on the tree (marked in green) and were determined as likely reinfections.

There were also isolates that mapped closely to other patient isolates on the tree, and these merited closer attention to see if there were genuine connections or unexpected problems caused by possible laboratory handling errors.

Panels b and c in Fig. 2 show one class of pattern that was observed with clustered isolate pairs, in which there were no SNP differences between each member of a pair, but each pair was very closely related to another pair. In both panels, the two pairs of samples came from different centres (panel b: 005 and 014, Harare and Marondera, both in Zimbabwe; panel c: 008 Harare, Zimbabwe, and 001 Francistown, Botswana, on the borders of Zimbabwe; Table 2), suggesting that a laboratory processing error was unlikely. An alternative explanation is that highly similar local strains were circulating in the two relatively close regions and had evolved independently over time.

**Table 2:** Relationship between single-nucleotide polymorphisms and mycobacterial interspersed repetitive units-variable number tandem repeat differences

Study number	Location	Treatment arm	SNPs	MIRU-VNTR differences	MIRU-VNTR loci amplified	Prediction
001	Francistown	4 month	0	0	21	Relapse
003	Harare	4 month	0	0	14	Relapse
005	Harare	4 month	0	0	20	Relapse
007	Harare	4 month	0	0	7	Relapse
008	Harare	4 month	0	0	11	Relapse
013	Marondera	4 month	0	0	10	Relapse
014	Marondera	4 month	0	0	11	Relapse
016	Johannesburg	4 month	0	0	14	Relapse
020	Johannesburg	4 month	0	0	17	Relapse
023	Cape Town	4 month	0	0	15	Relapse
029	Cape Town	4 month	0	0	2	Relapse
030	Cape Town	4 month	0	0	15	Relapse
032	Cape Town	4 month	0	0	14	Relapse
017	Johannesburg	6 month	0	0	17	Relapse
034	Cape Town	6 month	0	0	21	Relapse
037	Cape Town	Control	0	1	17	Relapse
011	Harare	4 month	0	1	16	Relapse
021	Johannesburg	4 month	0	1	16	Relapse
028	Cape Town	4 month	0	1	15	Relapse
024	Cape Town	Control	1	0	18	Relapse
033	Cape Town	Control	1	0	15	Relapse
010	Harare	4 month	1	0	18	Relapse
012	Harare	4 month	1	0	19	Relapse
025	Cape Town	4 month	1	0	11	Relapse
027	Cape Town	6 month	1	0	13	Relapse
019	Johannesburg	Control	1	2	4	Relapse
026	Cape Town	4 month	2	0	18	Relapse
002	Harare	4 month	3	0	15	Relapse
006	Harare	4 month	3	0	12	Relapse
018	Johannesburg	Control	5	0	5	Relapse
036	Cape Town	4 month	5	0	17	Relapse
031	Cape Town	6 month	5	0	16	Relapse
015	Johannesburg	Control	1294	7	14	Reinfection
035	Cape Town	4 month	57^a^	-	-	Relapse
004	Harare	Control	737	6	6	Reinfection
009	Harare	4 month	1233	3	3	Reinfection

The 36 isolates for which whole-genome sequencing was carried out are listed. With the mixed infection, although we concluded it to be a relapse, we could not precisely determine the SNP difference. For an explanation of the treatment arms, see the “Methods” section “RIFAQUIN trial” and Jindani et al. [17]. The table is sorted (in order) by number of SNPs, MIRU-VNTR differences, treatment arm and study number. Isolate 004-2 had previously been shown by Drug Susceptibility Testing (DST) to be resistant to isoniazid, rifampicin, ethambutol, streptomycin and pyrazinamide; however, all other isolates had been determined to be susceptible [17].

*MIRU-VNTR* mycobacterial interspersed repetitive units-variable number tandem repeats, *SNP* single-nucleotide polymorphisms

^a^It was not possible to separate the mixed genotypes to precisely determine a SNP difference

Panels d and e in Fig. 2 show a different type of pattern, in which a pair of isolates from one patient clustered together, as expected for relapses, but was also identical to a single isolate from another pair, suggesting a possible transmission event. In Fig. 2d, a post-treatment sequence for isolate 009 was identical to isolate pair 012; the two 009 isolates differed by 1233 SNPs. In Fig. 2e, a pre-treatment isolate 004-1 was identical in sequence to both isolates of patient 003; the two 004 isolates differed by 737 SNPs. All four patients received treatment in the same city, Harare (Table 2). While it is not impossible that these genotypes were genuinely isolated from the two patients, 009 and 004, another possible explanation is some form of laboratory processing error. Indeed, in one case the patients visited the hospital on the same day, and in the other results were reported at the same time. This combined with their geographical co-location would further support the possible processing error interpretation. It is also worth noting that if these are indeed errors, they would normally be invisible to the analysis without the resolution of WGS.

### Mixed infections

**Fig. 3:**
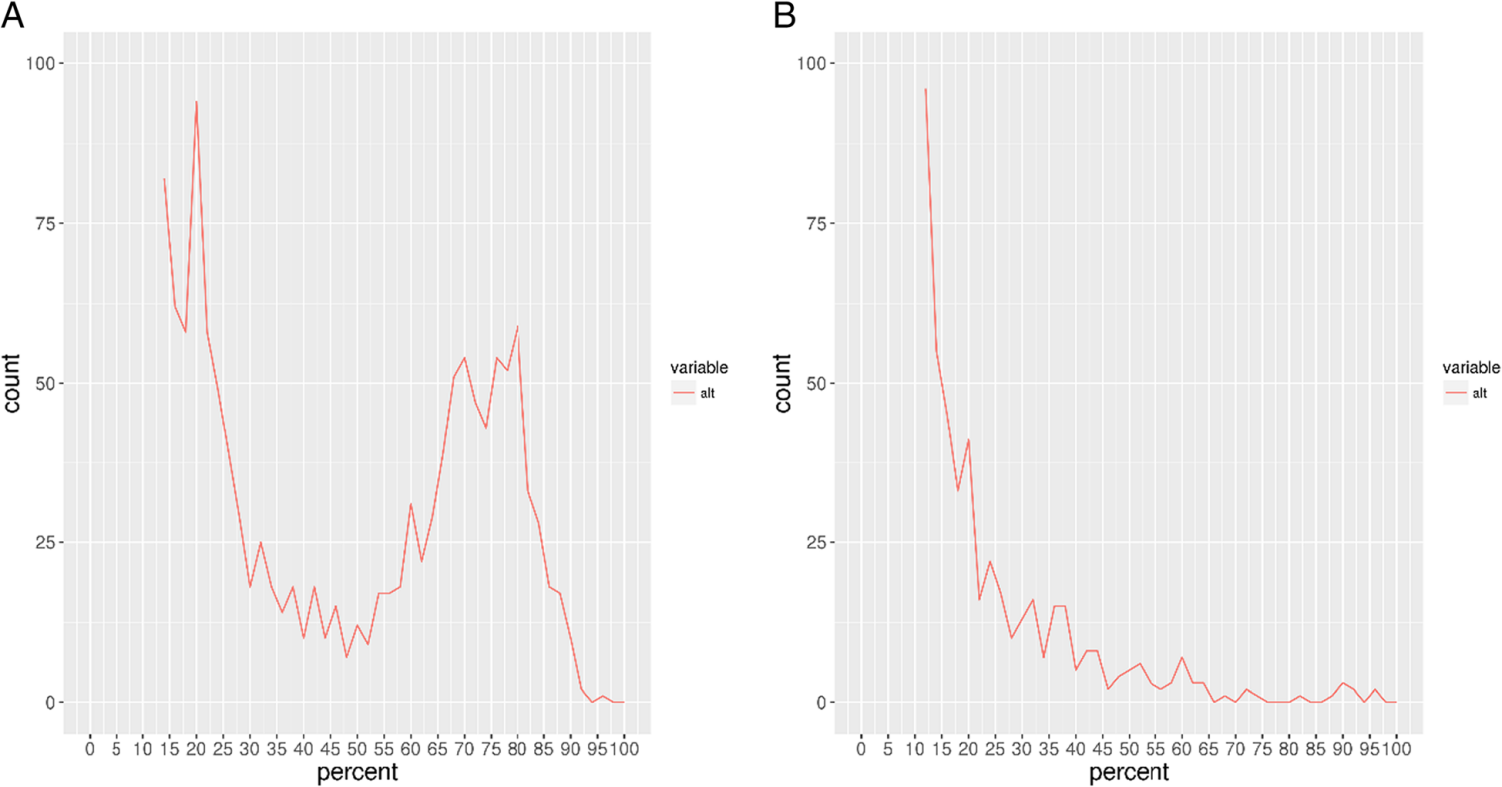
Identification of mixed infection. **a** Counts of genome sites which were called as a reference base but showed a significant proportion of sequence reads also supporting a variant base call (035-1); **b** the equivalent plot for an isolate with no mixed infection (035-2). The presence of a second peak in a is suggestive of a mixture with a minority genotype

One patient’s pair of samples (035) displayed 57 SNPs between the pre- (035-1) and post-treatment (035-2) isolates and was therefore initially classified as a reinfection. However, further analysis of the WGS data showed evidence of a mixed infection in the pre-treatment isolate (035-1; Fig. 3a) corresponding to an approximately 75% to 25% combination of two genotypes. Using this majority/minority ratio of read coverage, it was possible to separate the two genotypes and further phylogenetic reconstruction suggested that it was likely that the minority genotype (035-1-min) was closely related to the post-treatment isolate (035-2; Figs. 2a and 3b). This suggests that this was in fact a relapse of a previously unidentified minority genotype, rather than a reinfection as previously assigned.

Initially there appeared to be 57 SNP differences between the pre- and post-treatment isolates (035-1, 035-2), which would have been an unusual result given that the previous studies had only identified reinfections with very high SNP differences, and nothing at an intermediary level. The observation of mixed genotypes would explain this discrepancy because one of the main filtering criteria in the site-calling algorithm is to remove sites with mixed genotype calls (<75% read support for the call), so the real number of SNP differences between the isolates is likely to be higher. After separating the genotypes, it was estimated that the number of SNP differences between the pre-treatment minority genotype and the post-treatment isolates was 869 SNPs. The pre-treatment minority genotype and the post-treatment isolate appeared to differ by 245 SNPs; however, the genotype separation algorithm used was relatively crude, with filtering based on parameter cut-offs, so it was not possible to completely separate the genotypes at all mixed genome sites, reflecting the overlapping shape of the two distributions (Fig. 3a). However, the proximity of their placement on the tree (Fig. 2a) suggests they are highly related and thus this patient’s disease was likely a relapse.

### Comparing WGS with MIRU-VNTR data

**Fig. 4:**
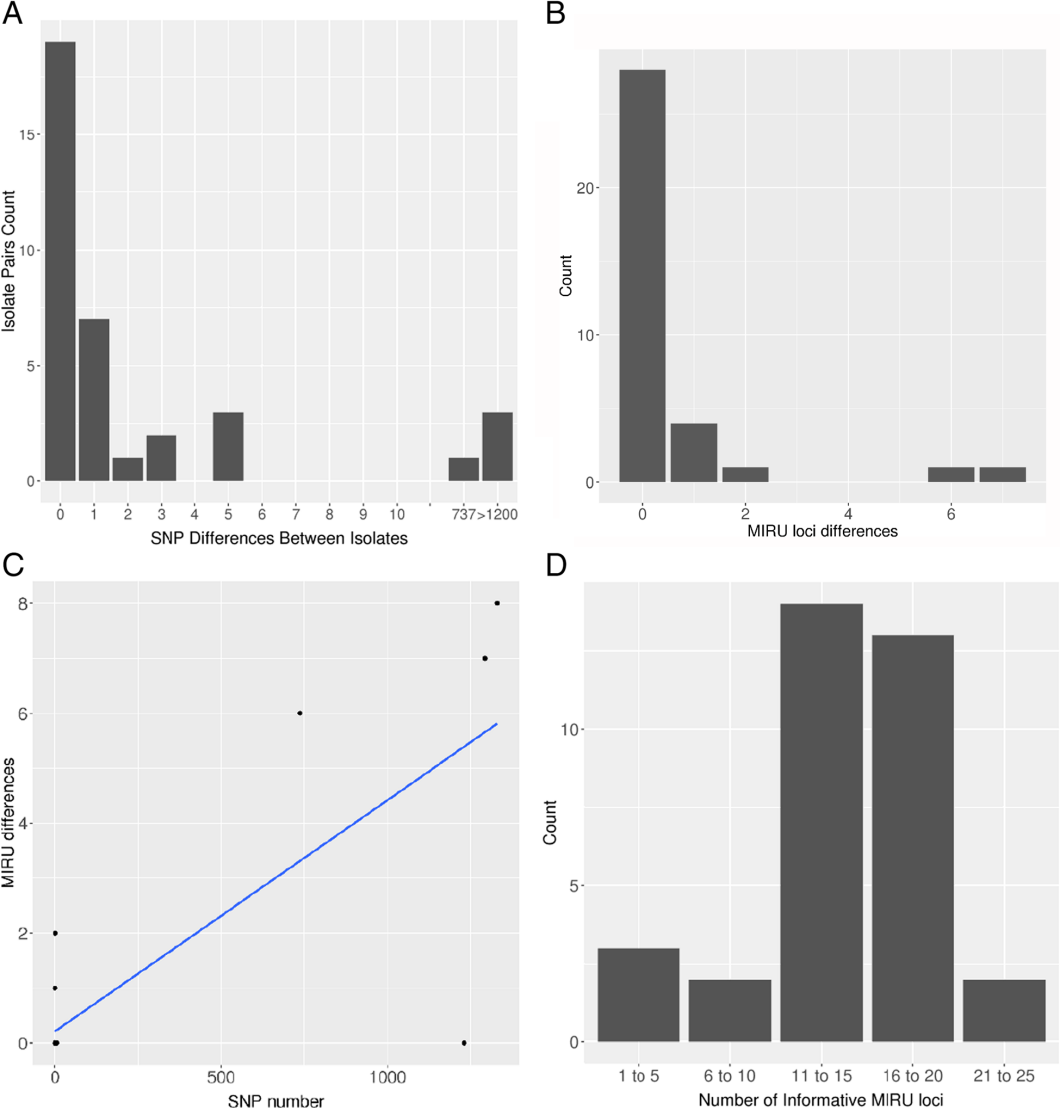
Analysis of single-nucleotide polymorphism (*SNP*) and mycobacterial interspersed repetitive units-variable number tandem repeat (*MIRU-VNTR*) differences between pairs of isolates. Data are summarised from Tables 1 and 2. **a** Number of SNP differences detected between paired isolates; **b** number of MIRU-VNTR differences detected between paired isolates; **c** correlation between SNP and MIRU differences; **d** number of informative MIRU loci on which differences were based (for each pair of samples, the lower number is shown)

Figure 4a shows there is a stark difference in the number of SNP differences between cases of relapse and reinfection, an observation also made by Bryant et al. [14]. Table 1 and Fig. 4b show the distribution of MIRU-VNTR differences. The majority of pairs had no MIRU-VNTR differences (out of up to 21 loci determined), but some had a maximum of seven loci different. We experienced technical difficulties which meant that the number of loci amplified varied (Table 2; see Discussion).

The relationship between SNP and MIRU-VNTR differences is shown in Table 2 and Fig. 4c. There was a clear MIRU-VNTR difference between those labelled as relapses using WGS (zero to two MIRU-VNTR differences) and those labelled as reinfections (seven to eight MIRU-VNTR differences). However, within the relapse group, there was no obvious relationship between these two measures: all samples with two to five SNPs had no MIRU-VNTR differences, whereas there were four with no SNP differences and one MIRU-VNTR difference. Overall, WGS largely agreed with MIRU-VNTR (Table 3), with only the likely mixed infection causing a possible discrepancy. That was based on a decision in the trial to classify pairs with two or more MIRU-VNTR differences as reinfections.

**Table 3:** Comparison of the use of whole-genome sequencing with mycobacterial interspersed repetitive units-variable number tandem repeats for calling relapse or reinfection

	MIRU-VNTR	WGS
Relapse	32	33
Reinfection	4	3

*MIRU* mycobacterial interspersed repetitive units-variable number tandem repeats, *WGS* whole-genome sequencing

### In silico spoligotyping and sub-lineages

Human *M. tuberculosis* strains have been divided into six global lineages, and further into sub-lineages, some of which may have distinct infection phenotypes [24]. In addition to the whole-genome SNP-based methodology used above, analysis using a set of 62 lineage-defining SNPs [24] was also used to assign sub-lineages (Additional file 1: Table S1). The three reinfections observed all involved different sub-lineages in the pair (patient 004: Euro-American LAM → Euro-American S type; patient 009: Euro-American S-type → East Asian; patient 015: Euro-American T → East Asian).

In silico spoligotyping was also performed (Additional file 1: Table S1). Of the 32 relapse pairs, 24 had identical spoligotypes and the remaining eight had one to seven spacer differences; all three reinfections had different spoligotypes (9–29 spacer differences).

### Antimicrobial resistance

Drug susceptibility testing showed that only one post-treatment isolate (004-2) had a drug-resistance phenotype, confirmed by genotyping (RIF^R^: *rpoB* S450L; INH^R^: *katG* S315T; EMB^R^: *embB* M306V), while its pre-treatment isolate partner (004-1) was susceptible to all drugs tested. Therefore, there was no evidence of any acquisition of antibiotic resistance during the trial in the samples that were tested with WGS.

### SNPs in relapse isolates

While most SNPs that arise in a strain between treatment and relapse would be expected to be random, as long as they are not deleterious, it would be a reasonable hypothesis that some SNPs may actively help the bacteria survive. Comparing the relapse pairs, 18 out of 30 SNPs were synonymous and 12 out of 30 were non-synonymous (Table 4). Of the 12 non-synonymous SNPs and two INDELs, none were in a gene associated with antibiotic resistance, in accord with the fact that no phenotypic resistance was seen. However, two SNPs lay in genes that are implicated in pathogenesis, both associated with *esx* Type 7 secretion systems (T7SSs) [25] (discussed below).

**Table 4:** Variants identified in relapse pairs

Strain pair	Type	Base number^a^	Gene	Function
002	NS	146316	*Rv0120c*, *fusA2*	Translation
	NS	345226	*Rv0283*, *eccB3*	Part of ESX-3 (essential, ESX-3 T7SS is implicated in metal homeostasis)
	S	3135592	*Rv2827c-109*	
	INDEL (TC/TCC)	3600992	*Rv3224B*	Predicted membrane protein
006	S	1348678	*Rv1205-41*	
	S	1370403	*Rv1227c*	
	S	2828233	*hisT down*	
010	NS	200390	*Rv0170*, *mce1B*	Part of ESX-1, essential for pathogenesis
012	NS	2510502	*Rv2237A*	CP, non-essential
017	INDEL (GC/GCC)	341124	*Rv0281*	Possible membrane protein
018	S	783720	*fusA1*	
	S	783729	*fusA1*	
	S	783732	*fusA1*	
	S	1476666	*rrl*	
	S	4050367	*folE*	
019	NS	3884906	*Rv3467*	CHP, non-essential
024	S	848538	*PPE12*	
025	S	1929374	*Rv1703c*	
026	NS	1192723	*Rv1069c*	CP, non-essential
	NS	1690758	*Rv1499*	CHP, non-essential
027	S	114494	*nrp*	
031	S	175753	*Rv0149*	
	S	620981	*Rv0530*	
	S	1315992	*pks4*	
	NS	1540497	*Rv1367c*	CP, non-essential
	S	2788333	*plsB2*	
033	NS	3618159	*Rv3240c, secA1*	Protein export, essential
036	S	923816	*lysT*	
	NS	924229	*Rvnt13, pheU*	tRNA
	NS	924234	*Rvnt13, pheU*	tRNA
	S	924263	*pheU*	
	NS	1476973	*Rvnr03, rrf*	5S rRNA

Function assigned using the Tuberculist database (http://tuberculist.epfl.ch/)

^a^SNPs between individual pairs predicted to be relapse

*CHP* conserved hypothetical protein, *CP* conserved protein, *NS* non-synonymous SNP, *S* synonymous SNP, *SNP* single-nucleotide polymorphism

## Discussion

### Relapse versus reinfection

In this study, high-quality genome sequence was generated for 36 pairs of isolates. The majority of pairs (32 of 36) were shown to have very few SNPs (≤5) between pre- and post-treatment *M. tuberculosis* isolates, suggestive of relapse and thus treatment failure.

On initial inspection, the other four pairs (4 of 36) had significant SNP differences between samples (57, 737, and two >1000), indicative of reinfection. However, phylogenetic analyses cast doubt on two pairs, in which a single isolate of each pair was highly related to another patient’s isolate in the study. While it is possible that these reflect transmission events, it is difficult to rule out some form of laboratory processing error; indeed, a transmission event so similar to another pair of samples in the trial (in one case the pre-treatment and in the other the post-treatment samples) would be relatively uncommon though not impossible, but such a pattern would be expected if there were a sample processing error and patient samples were swapped. A similar event was suggested by Casali et al. [26]. Indeed, trials inserting negative samples into the TB diagnostic process showed that errors can occur [27], but strain-typing methods allowed actual contamination to be detected. A review by Burman et al. [28] indicated a median false-positive rate of 3.1% in published studies. WGS can thus help identify when processing errors have occurred, thereby improving overall trial data quality and acting as a quality control measure of trial procedures.

The case with 57 SNP differences between the isolate pair was probably a mixed infection, and while accurate SNP figures could not be obtained, the data were consistent with a relapse from one of the two pre-existing strains. These are described as a major/minor strain within the sequencing data, but that may not accurately reflect the relative levels in the patient; these levels could, for example, be affected by colony size on the LJ slopes, and the actual loop sample taken for DNA preparation. The isolate pair were initially identified as being different from each other by a higher number of SNP differences (57) than would be expected for a relapse, but at an unusually low level of SNPs for a reinfection compared to other reported examples. This is likely to be due to the mixed infection causing many genuine SNPs to be discarded as uncertain by the site-calling algorithm. Reports of similar cases of mixed infections in previous studies [14, 15, 29] support the likelihood that this interpretation may be genuine, thus suggesting that it is important to assess isolates for evidence of mixed infections before calling relapse/reinfection.

Therefore, from the 36 pairs of isolates sequenced, there was strong evidence that 32 were relapses, one was a mixed infection masking a likely relapse, and three were reinfections, although two of these may have been the result of laboratory processing errors. This proportion (32 of 35 (91%) relapse: 3 of 35 (9%) reinfection; excluding the possible mixed infection) can be compared with previously reported relapse to reinfection proportions of 92:8, also in a chemotherapy trial [14], and 73:27 in the rather different situation of a long-term study with longer post-treatment follow-up (over 12 years in some cases) [15]. This latter study indicated that relapses occurred towards the start of the follow-up, and particularly within the first 2 years, and therefore is consistent with the study reported here.

### SNP differences in this and previous studies

The number of SNP differences in the relapse and reinfection groups was comparable to previous pre- and post-treatment studies (Table 5). Casali et al. [26] also found up to four SNP differences over 4 years in intra-patient studies. In each of the previous relapse studies, there was a large gap between the number of SNPs found in presumed relapses and in reinfections. This both lends support to the definition used to identify relapse versus reinfection, and also gives weight to the suggestion by Bryant et al. [14] that there is some immunity to reinfection by very similar strains. The same pattern was observed in this study, even though the phylogenetic tree showed that highly similar strains were circulating. Guerra-Assunção et al*.* [15] showed less SNP diversity in reinfections (100 rather than 1000 SNPs), and it would be interesting to determine if there is an effect of time, with similar strains only reinfecting after a longer passage of time. Casali et al. [26] demonstrated that there is strain diversity within a single sputum specimen, with up to 10 SNP differences seen when individual colonies were sequenced. The methodology described in this study deliberately took a sweep of colonies, which meant that much of this strain diversity within a single specimen would not be seen in WGS at the depth of coverage used.

**Table 5:** Number of single-nucleotide polymorphism differences between relapse and reinfection paired samples in different studies

Relapse group	Reinfection group	Maximum length of follow-up	Study
0–5	>1000	18 months	This study
0–6	>1300	18 months	Bryant et al. [14]
0–8	>100	>12 years	Guerra-Assunção et al. [15]

### SNPs seen in relapse isolates

For 16 of the 32 relapse pairs sequenced, SNPs were identified between the isolates (Table 4; excluding the mixed infection). While it is likely that many or most of these will not be advantageous to the bacteria, it is a plausible hypothesis that some of them might have a survival advantage.

Of the 12 non-synonymous SNPs observed in relapse isolate pairs, two were in gene systems that have proven involvement with pathogenesis: the two T7SSs *esx1* and *esx3*. One lay in *eccB3*, which is a gene in the ESX3 T7SS, which is essential for growth. This system is involved in pathogenesis, partly through the control of iron acquisition, which appears to have a role in metal homeostasis [30]. The other was located in *mce1B*, which is a gene in the ESX1 T7SS, which is essential for virulence and exports the well-characterised ESAT-6/CFP10 complex [25]. Bryant et al. [14] reported that two genes with SNPs had functions associated with oxidative stress, and Guerra-Assunção et al. [15] reported an association with *katG*, well known for being involved in resistance to both oxidative stress and isoniazid. Clearly these may just be chance associations, but they also indicate potential avenues for studying bacterial survival during chemotherapy. The scale of investment in phase 2 and 3 trials is such that there is an obligation to extract as much information as possible from the study and the contribution of WGS is fundamental to understanding the bacteriology under treatment.

### Mixed infections

A potential confounder in differentiating relapse from reinfection is that of mixed infections. If either the initial or subsequent infection is mixed, then sampling just one isolate could give a misleading designation. One likely mixed infection was identified with a 75:25 genotype ratio, although this ratio may not represent the ratio of the mixture in the bacterial population in vivo.

Of course, these methods would only reveal mixed infections with significant proportions of each strain, and it cannot formally exclude the possibility that other infections were also mixed, but at a very low levels. Bryant et al*.* [14], Guerra-Assunção et al. [15], Casali et al. [26] and Köser et al. [29] all identified mixed infections using WGS. Other studies have demonstrated them using alternative techniques, including MIRU-VNTR [31–34], but WGS is more powerful, and Bryant et al. [14] found that WGS detected more mixed infections than MIRU-VNTR.

The definition of a mixed infection is made less clear by the finding that at least 10 SNP differences can be found within a single sputum sample [26], and the observation that very similar strains circulate in high-prevalence settings (e.g. Fig. 2). However, the data here and in the previous relapse studies [14] suggest that some sort of immunological protection might exist that makes successful co-infection with a similar strain less likely.

### Comparing WGS to MIRU-VNTR and spoligotyping

Previously, owing to its speed and digital output, MIRU-VNTR has been preferred to the earlier IS*6110* profiling as a means of typing *M. tuberculosis* isolates; indeed, it was only recently described as “the new reference standard for molecular epidemiological studies” [35].

In this study, there was a correlation between SNP and MIRU-VNTR differences for isolates predicted to be cases of relapse (0–5 SNP; 0–2 MIRU-VNTR loci) and reinfection (SNP > 1000; MIRU-VNTR loci ≥7). This is in contrast with the study of Bryant et al. [14] who reported that three reinfection pairs had 1–13 different loci, although that study was an interim analysis performed prior to final data resolution and unbinding, which may have impacted on the ultimate assignment of the patients. Furthermore, Casali et al*.* [26] found that two MIRU-VNTR differences could correspond to a significant number of SNP differences. A transmission study by Walker et al. [11] only examined isolates with successful 24-loci MIRU-VNTR data, showing that, up to a difference of 100 SNPs, isolates could have 1–3 MIRU-VNTR locus differences, while above 100 SNP differences, the number of MIRU-VNTR changes increased.

Achieving consistent results with MIRU-VNTR, which involves 24 multiplexed PCRs, is known to be technically challenging [14, 26, 36, 37]. Indeed, there was significant variation in the number of loci amplified in this study (Table 2, Fig. 4d), which we attribute to a combination of DNA quantity and quality, and the technical difficulties referred to above. Furthermore, other limitations and issues with MIRU-VNTR in relation to the study setting have been discussed in a systematic review [38].

WGS is technically more straightforward and was comparable in cost in our hands (~£100 per sample), but with reducing costs and whole-genome resolution, it is clearly a superior, more robust method then MIRU-VNTR for strain typing. In addition, WGS can provide additional information by identifying markers associated with drug resistance, which could be useful in the context of relapsing cases in a clinical trial. Sequence data is also more amenable to incorporation into other studies and will provide further information on TB evolution as global databases of genome information grow.

Spoligotyping has been widely used for robust division of *M. tuberculosis* into different sub-types [4], but we found that SNPs were not only far more sensitive for determining relapses and reinfections, but also more useful for assigning sub-lineages.

### Value of WGS in chemotherapy trials

The data from this study in combination with the previous two relapse studies [14, 15] allow an evaluation of the relative benefit of using WGS or MIRU-VNTR as a means of determining relapses from reinfections in chemotherapy trials.

The RIFAQUIN trial was in an area of high endemicity, suggesting that reinfections are not likely to be higher elsewhere due to disease prevalence. Thus, the data presented in this study and previously [14, 15] indicate that the proportion of reinfections is very low compared to relapse, although Guerra-Assunção et al. [15] suggest that reinfections may rise at later time points after completion of therapy. Furthermore, cases in which isolates are identified as reinfections are more likely to be wrong, because the possible errors observed here (processing errors, unrecognized mixed infections) are more likely to suggest a reinfection.

## Conclusions

In the pre-genomic era, all post-treatment infections were presumed to be relapses, and it could be argued that, due to the low reinfection rates and the increased cost and time required to perform the sequencing, WGS provides only modest gains for the analysis of the primary outcome in a chemotherapy clinical trial of this nature.

Nevertheless, in addition to robust genomic evidence for treatment outcome, the added information that WGS provides is scientifically valuable and will become of greater value as more genome sequence data and more information about the genotype–phenotype correlation and its impact on disease and transmission becomes available. Furthermore, future trials for new TB drugs in the development pipeline or novel combination regimens may be held in areas of high TB prevalence where re-infection or mixed infections are more likely, thus making accurate strain discrimination imperative; in these instances, WGS should be the method of choice.

## Additional file

Additional file 1: Table S1. Spoligotype and sub-lineage information for isolates. (DOCX 23 kb)
