# Supplementary material for: Use of whole-genome sequencing to distinguish relapse from reinfection in a completed tuberculosis clinical trial
Source: BMC Med. 2017 Mar 29;15:71. doi: 10.1186/s12916-017-0834-4 (PMC5371199; doi:10.1186/s12916-017-0834-4)
Supplement: Additional file 1: Table S1. — Spoligotype and sub-lineage information for isolates. (DOCX 23 kb) [file 12916_2017_834_MOESM1_ESM.docx]

**Additional file 1**

Table S1 Spoligotype and sub-lineage information for isolates. **^a^** class predicted as relapse, reinfection, or mixed (see text and Figure 2 for details). ^b^ Sub-lineages derived from spoligotypes as listed in SITVITWEB database (1). ^c^ sub-lineages derived from 62 lineage defining SNPs defined by Coll *et al* (2).

**References**

1. Demay C, Liens B, Burguière T, Hill V, Couvin D, Millet J, et al. SITVITWEB – A publicly available international multimarker database for studying Mycobacterium tuberculosis genetic diversity and molecular epidemiology. Infect Genet Evol. 2012 Jun;12(4):755–66.

2. Coll F, McNerney R, Guerra-Assunção JA, Glynn JR, Perdigão J, Viveiros M, et al. A robust SNP barcode for typing Mycobacterium tuberculosis complex strains. Nat Commun. 2014 Sep 1;5:4812.

| **Id** | **class ^a^** | **spoligotype** | **Sub-lineage ^b^** | **SIT ^b^** | **SNP sub-lineage ^c^** | **SNP sub-lineage ^c^** |
| --- | --- | --- | --- | --- | --- | --- |
| 001-1 | relapse | 777777777760771 | T1 | 53 | lineage4.8 | Euro-American (mainly T) |
| 001-2 | relapse | 777777777760771 | T1 | 53 | lineage4.8 | Euro-American (mainly T) |
| 002-1 | relapse | 677777605560771 | T1a |  | lineage4.3.3 | Euro-American (LAM) |
| 002-2 | relapse | 677777605560771 | T1a |  | lineage4.3.3 | Euro-American (LAM) |
| 003-1 | relapse | 757777606060771 | LAM11-ZWE | 1466 | lineage4.3.4.2.1 | Euro-American (LAM) |
| 003-2 | relapse | 757777606060771 | LAM11-ZWE | 1466 | lineage4.3.4.2.1 | Euro-American (LAM) |
| 004-1 | reinfection | 757777606060771 | LAM11-ZWE | 1466 | lineage4.3.4.2.1 | Euro-American (LAM) |
| 004-2 | reinfection | 776377777760771 | S | 34 | lineage4.4.1.1 | Euro-American (S-type) |
| 005-1 | relapse | 700076777760771 | X3 | 92 | lineage4.1.1.3 | Euro-American (X-type) |
| 005-2 | relapse | 700076777760771 | X3 | 92 | lineage4.1.1.3 | Euro-American (X-type) |
| 006-1 | relapse | 703777740003171 | CAS1-Delhi | 25 | lineage3 | East-African-Indian |
| 006-2 | relapse | 703777740003171 | CAS1-Delhi | 25 | lineage3 | East-African-Indian |
| 007-1 | relapse | 000000000003771 | Beijing | 1 | lineage2.2.1 | East-Asian |
| 007-2 | relapse | 000000000003771 | Beijing | 1 | lineage2.2.1 | East-Asian |
| 008-1 | relapse | 777777777760771 | T1 | 53 | lineage4.8 | Euro-American (mainly T) |
| 008-2 | relapse | 777777777760771 | T1 | 53 | lineage4.8 | Euro-American (mainly T) |
| 009-1 | reinfection | 436377777760771 |  |  | lineage4.4.1.1 | Euro-American (S-type) |
| 009-2 | reinfection | 000000000003771 | Beijing | 1 | lineage2.2.1 | East-Asian |
| 010-1 | relapse | 702777740003771 | CAS1-Delhi | 1092 | lineage3 | East-African-Indian |
| 010-2 | relapse | 702777740003771 | CAS1-Delhi | 1092 | lineage3 | East-African-Indian |
| 011-1 | relapse | 777777777760771 | T1 | 53 | lineage4.9 | Euro-American (H37Rv-like) |
| 011-2 | relapse | 777777777760771 | T1 | 53 | lineage4.9 | Euro-American (H37Rv-like) |
| 012-1 | relapse | 000000000003771 | Beijing | 1 | lineage2.2.1 | East-Asian |
| 012-2 | relapse | 000000000003771 | Beijing | 1 | lineage2.2.1 | East-Asian |
| 013-1 | relapse | 700076777760771 | X3 | 92 | lineage4.1.1.3 | Euro-American (X-type) |
| 013-2 | relapse | 700076777760771 | X3 | 92 | lineage4.1.1.3 | Euro-American (X-type) |
| 014-1 | relapse | 700076777760771 | X3 | 92 | lineage4.1.1.3 | Euro-American (X-type) |
| 014-2 | relapse | 700076777760771 | X3 | 92 | lineage4.1.1.3 | Euro-American (X-type) |
| 015-1 | reinfection | 777777777760700 | T1 | 51 | lineage4.7 | Euro-American (mainly T) |
| 015-2 | reinfection | 000000000003671 | Beijing | 255 | lineage2.2.1 | East-Asian |
| 016-1 | relapse | 777777777760771 | T1 | 53 | lineage4.9 | Euro-American (H37Rv-like) |
| 016-2 | relapse | 567777777760771 |  |  | lineage4.9 | Euro-American (H37Rv-like) |
| 017-1 | relapse | 000000000000000 | ATYPIC | 2669 | lineage4.3.3 | Euro-American (LAM) |
| 017-2 | relapse | 000000000000000 | ATYPIC | 2669 | lineage4.3.3 | Euro-American (LAM) |
| 018-1 | relapse | 500076777760331 |  |  | lineage4.1.1.3 | Euro-American (X-type) |
| 018-2 | relapse | 700076757760771 | X3 | 549 | lineage4.1.1.3 | Euro-American (X-type) |
| 019-1 | relapse | 777776717760771 |  |  | lineage4.1.1.2 | Euro-American (X-type) |
| 019-2 | relapse | 777776717760771 |  |  | lineage4.1.1.2 | Euro-American (X-type) |
| 020-1 | relapse | 000000000003771 | Beijing | 1 | lineage2.2.1 | East-Asian |
| 020-2 | relapse | 000000000003771 | Beijing | 1 | lineage2.2.1 | East-Asian |
| 021-1 | relapse | 100076767560510 |  |  | lineage4.1.1.3 | Euro-American (X-type) |
| 021-2 | relapse | 700076777760771 | X3 | 92 | lineage4.1.1.3 | Euro-American (X-type) |
| 022-1 | relapse | 000000000003771 | Beijing | 1 | lineage2.2.1.1 | East-Asian |
| 022-2 | relapse | 000000000003771 | Beijing | 1 | lineage2.2.1.1 | East-Asian |
| 023-1 | relapse | 000000000003771 | Beijing | 1 | lineage2.2.1 | East-Asian |
| 023-2 | relapse | 000000000003771 | Beijing | 1 | lineage2.2.1 | East-Asian |
| 024-1 | relapse | 776377777760771 | S | 34 | lineage4.4.1.1 | Euro-American (S-type) |
| 024-2 | relapse | 776377777760771 | S | 34 | lineage4.4.1.1 | Euro-American (S-type) |
| 025-1 | relapse | 700036777560461 |  |  | lineage4.1.1.3 | Euro-American (X-type) |
| 025-2 | relapse | 700076777760771 | X3 | 92 | lineage4.1.1.3 | Euro-American (X-type) |
| 026-1 | relapse | 770000777760771 | T1-RUS2 | 280 | lineage4.8 | Euro-American (mainly T) |
| 026-2 | relapse | 770000777760771 | T1-RUS2 | 280 | lineage4.8 | Euro-American (mainly T) |
| 027-1 | relapse | 000000007760771 |  | 4 | lineage4.3.2.1 | Euro-American (LAM) |
| 027-2 | relapse | 000000007720771 | H3 | 3 | lineage4.3.2.1 | Euro-American (LAM) |
| 028-1 | relapse | 776177607760771 | LAM3 | 33 | lineage4.3.2.1 | Euro-American (LAM) |
| 028-2 | relapse | 776177607760771 | LAM3 | 33 | lineage4.3.2.1 | Euro-American (LAM) |
| 029-1 | relapse | 700016777760771 | X3 | 2020 | lineage4.1.1.3 | Euro-American (X-type) |
| 029-2 | relapse | 700016777760771 | X3 | 2020 | lineage4.1.1.3 | Euro-American (X-type) |
| 030-1 | relapse | 777777747760771 | T1 | 1580 | lineage4.1.2.1 | Euro-American (Haarlem) |
| 030-2 | relapse | 777777747760771 | T1 | 1580 | lineage4.1.2.1 | Euro-American (Haarlem) |
| 031-1 | relapse | 000000000003771 | Beijing | 1 | lineage2.2.1 | East-Asian |
| 031-2 | relapse | 000000000002711 |  |  | lineage2.2.1 | East-Asian |
| 032-1 | relapse | 777776777760771 | X1 | 119 | lineage4.1.1.3 | Euro-American (X-type) |
| 032-2 | relapse | 777776777760771 | X1 | 119 | lineage4.1.1.3 | Euro-American (X-type) |
| 033-1 | relapse | 776370001760771 | S | 1333 | lineage4.4.1.1 | Euro-American (S-type) |
| 033-2 | relapse | 776370001760771 | S | 1333 | lineage4.4.1.1 | Euro-American (S-type) |
| 034-1 | relapse | 000000000003771 | Beijing | 1 | lineage2.2.1.1 | East-Asian |
| 034-2 | relapse | 000000000003751 | Beijing | 941 | lineage2.2.1.1 | East-Asian |
| 035-1-min | mixed | not done |  |  |  |  |
| 035-1-maj | mixed | not done |  |  |  |  |
| 035-2 | mixed | 577777377760631 |  |  | lineage4.8 | Euro-American (mainly T) |
| 036-1 | relapse | 773174346720471 |  |  | lineage4.1.2 | Euro-American |
| 036-2 | relapse | 777777347760471 | T4-CEU1 | 39 | lineage4.1.2 | Euro-American |
